# Health challenges of older women following attempted intimate partner homicide

**DOI:** 10.1093/geront/gnaf287

**Published:** 2025-12-08

**Authors:** Hila Avieli

**Affiliations:** Department of Criminology, Ariel University, Ariel, Israel

**Keywords:** Older women, Intimate partner homicide, Qualitative study, Intersectionality, Health

## Abstract

**Background and Objectives:**

While some research exists on women who have survived attempted intimate partner homicide (IPH), studies focusing on survivors aged 60 years and older and how they cope with health concerns remain scarce. Guided by intersectionality as a theoretical framework, this study examined older women’s experiences of managing their health in the aftermath of surviving a violent attack by a male partner, considering how intersecting identities such as age, gender, and survivorship shape their health-related challenges. The insights gained could inform targeted interventions and policies to address this population’s unique vulnerabilities and care needs.

**Research Design and Methods:**

Interpretative phenomenological analysis methodology was used to conduct in-depth, semi-structured interviews with nine older women who had survived an attempted IPH. A purposeful sampling strategy was employed to identify women who had been legally or medically classified as survivors of attempted intimate partner homicide and self-identified as such. The interviews, guided by an interview guide, were recorded, transcribed verbatim, and analyzed to elicit the main themes.

**Results:**

Three themes emerged from the participants’ narratives: (a) the ripple effect of the injury, (b) reduced access to formal and informal health-related support systems, and (c) limited financial resources for recovery needs.

**Discussion and Implications:**

The interplay of chronic health conditions, minimal support networks, and financial constraints highlights how advancing age, traumatic histories, and systemic healthcare gaps are mutually exacerbating. These findings underscore the value of integrated, age-conscious, and trauma-informed services and theoretical frameworks that address the complete spectrum of older survivors’ healthcare needs.

## Background

Intimate partner homicide (IPH), defined as the intentional killing of a current or former partner (Kivisto, 2015), is the leading form of homicide against women worldwide (UNODC, 2019). Although research suggests that IPH among older women may follow distinct patterns, the study of this population remains limited (Benbow et al., 2019; Salari, 2007). When perpetrators fail to carry out their intention to kill, survivors may be perceived as victims of attempted intimate partner homicide, also called “failed femicide,” “attempted femicide,” “near-lethal violence,” and “attempted homicide against women” (Abrunhosa et al., 2021; Cantor et al., 2022; Vatnar & Bjørkly, 2013; Weil, 2016). These survivors’ experiences have received little scholarly attention despite evidence of unique relationship dynamics leading up to the attempted IPH, as well as subsequent emotional, social, and financial hardships (Avieli, 2025b; Weil, 2016). Circumstances of older survivors of attempted IPH remain largely unexplored. This study addressed this gap by investigating the experiences of older women survivors of attempted IPH perpetrated by male partners or ex-partners, focusing particularly on how they perceived and managed their health in this stage of life.

### Intimate partner homicide among older women

While existing research tends to focus on younger women in IPH studies (Breitman et al., 2004), older women, commonly defined as aged 60 years and above (Benbow et al., 2019; Bows, 2019), account for approximately 17% of all female victims of intimate partner or family-related homicide (UNODC & UN Women, 2024). However, this figure likely underrepresents the true scope of the issue, as older women remain significantly underrepresented in IPH research (Kennedy et al., 2023). This underrepresentation is further compounded by widespread underreporting and misclassification in official data (e.g., attributing deaths to health-related causes or aggregating cases that obscure distinctions between younger and older IPH victims) (Benbow et al., 2019; Bows, 2019; Salari & Maxwell, 2016). Thus, the unique context and factors surrounding IPH in older women are obscured (Weil & Keshet, 2021).

Nonetheless, several distinct characteristics have emerged from the limited research on this population. For example, studies point to a higher prevalence of homicide–suicide incidents among older than younger couples (MacPherson et al., 2020; Salari & Sillito, 2016). One contributing factor is the concealment of prolonged abuse, as older generations’ societal norms often discourage victims from seeking help (Bourget et al., 2010; MacPherson et al., 2020). In addition, financial and legal disputes, such as conflicts over shared assets or inheritance, can further escalate tensions and increase the risk of lethal violence (Benbow et al., 2019). Health challenges and caregiving responsibilities may add strain to relationships, intensifying existing conflicts (Roberto et al., 2013; Salari, 2007). Social isolation further compounds the issue by making it more difficult for outsiders to either detect signs of abuse or intervene before violence occurs (MacPherson et al., 2020).

These insights highlight the importance of deepening the understanding of IPH among older women as a distinct phenomenon (Benbow et al., 2019; Bows, 2019). One way to increase this knowledge is by listening to survivors’ voices—women who survived attempted IPH.

### Survivors of intimate partner homicide

Research on women who survived an attempted IPH remains scarce (Weil, 2016). Existing studies focus primarily on precursors to attempted homicide (Harden et al., 2019; Nicolaidis et al., 2003; Santucci, 2021), often revealing common warning signs such as psychological abuse, jealousy, threats, stalking, controlling behavior, normalized violence, and reluctance to seek help (Evans et al., 2018; Farr, 2002; Harden et al., 2019; Nicolaidis et al., 2003; Santucci, 2021; Weil, 2016). A few studies addressed survivors’ post-incident experiences, noting emotional entrapment, fear, and altered behaviors (Avieli, 2025a; Thomas et al., 2014) alongside financial, occupational, and housing instability (Farr, 2002).

Within this limited body of scholarship, the age of attempted IPH survivors has not been addressed; in particular, older women’s experiences of survivorship are currently missing from the discourse. The little information available about older women who survived an attempted IPH is typically from studies on attempted IPH survivors of all ages, when samples included older participants in addition to younger ones. For example, Azevedo et al. (2015) studied three IPH survivors, including a 57-year-old who endured decades of abuse marked by control, submission, and isolation prior to the attempted IPH. The authors noted how societal norms and traditional gender roles reinforced the participant’s subservient position, which prolonged the abuse, and how, after surviving the IPH attempt, she struggled to rediscover her identity and live independently for the first time following years of dependence. In a study by Vatnar and Bjørkly (2013), 4 of the 157 participants were over 60 years old. Although these older participants were part of the sample, the study did not specifically analyze their experiences or draw age-specific conclusions.

Santucci (2021) noted that participants’ ages ranged up to 75 years but did not state older adults’ precise ages. In addition, no substantial age-related differences in experiences of abuse or attempted IPH were reported. Similarly, Nicolaidis et al. (2003), Vella et al. (2017), and Harper (2022) examined female IPH survivors, with the oldest participants being 54, 61, and 64 years old, respectively. However, because these studies did not aim to focus on participants’ ages, they neither collected nor analyzed detailed age-related data, nor did they draw any conclusions specifically addressing older adult women in their samples.

### Health challenges faced by severe intimate partner vioelnce and attempted intimate partner homicide survivors

The health consequences of severe physical IPV (intimate partner violence) and attempted IPH encompass both immediate and long-term outcomes. Acute physical outcomes such as fractures, traumatic brain injuries, and musculoskeletal damage are well-documented (Alessandrino et al., 2020; Thomas et al., 2021). Beyond these initial injuries, survivors often face chronic health conditions including persistent pain, fibromyalgia, gastrointestinal issues, and increased risk for metabolic and cardiovascular disorders. These outcomes are frequently mediated by stress-related physiological responses, behavioral disruptions, and limited access to timely and adequate healthcare (Stubbs & Szoeke, 2022; Wuest et al., 2010). In addition to physical effects, survivors frequently experience psychological challenges such as post-traumatic stress disorder, anxiety, and depression, which can further complicate recovery and interfere with health management (Wuest et al., 2009; Best et al., 2021).

Attempted IPH, while overlapping with IPV in many ways, often results in particularly violent and life-threatening injuries. Survivors may endure stab or gunshot wounds, and other forms of extreme trauma requiring surgery, hospitalization, and long-term rehabilitation (Farr, 2002; Nicolaidis et al., 2003; Santucci, 2021). In the aftermath of such violence, many women report ongoing secondary health issues, including reduced mobility, chronic pain, respiratory problems, and sleep disturbances (Santucci, 2021; Thomas et al., 2014). These physical challenges are compounded by significant emotional distress, as survivors often experience heightened trauma symptoms that further hinder both emotional and physical recovery (Thomas et al., 2014; Weil, 2016).

These consequences may be particularly acute among older women, who often endure abusive situations with preexisting health conditions and age-related vulnerabilities. Factors such as diminished physiological resilience, chronic illness, cognitive decline, and social isolation may intensify the impacts of IPV and attempted IPH, slowing recovery and deepening harm (Mouton, 2003; Pathak et al., 2019). For older survivors, severe violence can accelerate physical deterioration and cognitive impairment, while simultaneously contributing to new psychological and emotional difficulties (Stubbs & Szoeke, 2022). Moreover, older women may face systemic obstacles when attempting to access support, including ageist attitudes from health professionals, a lack of trauma-informed care, and fragmented service delivery, all of which can undermine recovery and well-being over time (Pathak et al., 2019). Despite growing recognition of the long-term health impacts of IPV, research specifically addressing older women who survive attempted IPH remains extremely limited.

### Intersectionality as a theoretical framework for studying the experiences of older intimate partner homicide survivors

This study used intersectionality to explore experiences of older women who survived attempted IPH, emphasizing how interconnected social factors, such as age and gender, shape identities and influence lived experiences (Gaber et al., 2025; Rahman, 2008). Thus, the experience of being an “older woman who survived attempted IPH” is distinct from merely being “an older person,” “a woman,” and “a survivor” (Sun et al., 2018). A single-axis view (e.g., only age or only gender oppression) fails to capture this complexity, which emerges from the interplay of multiple marginalized or privileged identities (Crenshaw, 1989; Hill Collins, 2009). Indeed, some scholars suggest that using an intersectional lens—incorporating identities such as race, class, and gender alongside forms of oppression such as racism, sexism, and classism—offers a more nuanced view of IPH by illuminating diverse experiences and systemic factors (Cullen et al., 2021; Graham et al., 2022; Sosa, 2023). In this context, Harper (2017) shows how overlapping race, class, and gender oppressions—such as structural poverty and distrust of law enforcement—trap Latina women in lethal relationships and reduce their likelihood of self-help. Similarly, Posey (2024) and Gray et al. (2024) highlight how firearms access, cultural stigma, and institutional barriers intersect to discourage help-seeking and heighten IPH risk.

While studies using intersectionality as a theoretical framework often examine the intersections of race, class, and other socioeconomic factors, intersectionality as a theoretical framework has also been applied to explore more specific identity intersections, focusing primarily on dimensions such as age and marginalization (Avieli & Band-Winterstein, 2024), gender and disability (Saxe, 2017), or gender and institutional position (Fay et al., 2021). In this context, this study highlights older age and intimate partner victimization as central to the participants’ experiences of exclusion.

Thus, the aim of this study was to understand, through an intersectional lens, how older women who survived an attempted IPH navigate health challenges and address their recovery needs. Based on this aim, the guiding research question was as follows: How do older women who have survived an attempted IPH perceive their experiences of navigating health challenges, and how do they understand their recovery needs?

## Research design and methods

This study used interpretive phenomenological analysis (IPA) methodology (Smith et al., 2009) to explore the participants’ experiences. By focusing on how individuals experience and interpret their world, this qualitative method offers rich insights into the intricate emotional and psychological elements at play (Pietkiewicz & Smith, 2014). This study was part of a larger research project investigating survivors of IPH.

### Participants and sample

Purposeful sampling (Patton, 2015) was used to select nine women who met the following criteria: (a) aged 60 years and above at the time of the violent attack; (2) had been violently attacked by a partner (either husband or boyfriend) with the intent to kill or, at least, to cause life-threatening injuries; (c) had been identified as an attempted murder case at some point during legal proceedings or medical care; (d) considered themselves survivors of attempted IPH.

The participants’ ages were between 60 and 75 years (*M *= 68.3). No standardized age cutoff exists for defining older women in IPH contexts (Weil & Keshet, 2021), so the cutoff age of 60 years was determined in accordance with other research on this population (Benbow et al., 2019; Bows, 2019). All participants sustained physical injuries during the attempted IPH and required hospitalization. After discharge, they continued to receive medical care from local doctors’ offices and community clinics. See online supplementary material, Appendix A for additional sociodemographic information.

### Data collection

Data were collected using semi-structured phenomenological interviews based on an interview guide (Pietkiewicz & Smith, 2014), including the following broad categories: the attempted IPH and its immediate health consequences (e.g., What do you remember from the hours and days following the violent incident? How did you feel physically and emotionally while still in the hospital? What were short-term consequences of the violent attack?), the experience of receiving medical care following attack (e.g., Can you describe the kind of medical care you require now and how you obtain it? How do you feel about this ongoing healthcare?), and a retrospective view of the way the attempted IPH has impacted health (e.g., What were long term consequences of the violent attack? Thinking about your physical well-being over the years, how do you feel the incident has influenced any challenges or difficulties you face today?). Please see online supplementary material, Appendix B for the complete interview guide.

### Procedure

Individuals were selected and engaged in interviews following guidelines for research on sensitive issues (Liamputtong, 2007). The researcher is a criminologist and social worker with experience in exploring sensitive research topics and interviewing marginalized populations.

After the University’s Research Ethics Committee approved the study, participant well-being was prioritized by recruiting only women who had already spoken publicly about surviving an attempted IPH. Participants were located through social-media posts, newspaper articles, and television features. Limiting recruitment to self-disclosed survivors reduced the risk of intruding on those who had not processed or shared their experience and aligned with best-practice guidelines for sensitive research, which recommend contacting only individuals who have voluntarily come forward to minimize traumatization (Wolf et al., 2019). The researcher then contacted 12 women via telephone and explained the aim of the study, assessed the candidates’ compliance with the inclusion criteria, and addressed their concerns. Nine of these preliminary conversations led to face-to-face interviews held at the participant’s location of choice (in most cases, the participant’s home).

At the beginning of each interview, the participant signed a consent form, ensuring confidentiality and the freedom to end the interview without consequences. During the interviews, the researcher carefully observed participants’ emotional reactions and continuously sought their consent throughout the interview process (Kavanaugh & Ayres, 1998). Each interview lasted between 1.5 and 2.5 h, was audio-recorded, and transcribed verbatim. Additional measures to protect participants’ anonymity and confidentiality involved modifying identifying information and storing all data on a password-protected computer.

### Data analysis and trustworthiness

Data analysis followed the updated guidelines for IPA set out by Smith and Nizza (2022). First, multiple readings of the transcripts provided a holistic sense of the participants’ narratives. Exploratory commenting came next, annotating significant remarks from each interview, for example, a participant describing her injuries’ continued interference with daily activities. These annotations were then distilled into experiential statements that captured the essence of each person’s lived experience. The researcher repeatedly consulted the transcripts to confirm or refine these statements, consistent with the iterative process of IPA, identified connections across the data, and clustered related ideas into personal experiential themes. A deductive lens guided by intersectional theory was then used to explore how participants perceived the interplay between aging, physical health limitations, and post-IPH vulnerabilities. Throughout the process, the researcher engaged with colleagues to assess areas of convergence or divergence between participants’ accounts, ensuring that each emergent theme was grounded in individual narratives and reflective of broader patterns. Ultimately, these refined clusters formed three group experiential themes that represent the shared yet distinct dimensions of the participants’ experiences.

To establish trustworthiness, a series of measures was implemented: First, audio-recorded interviews, along with their verbatim transcriptions, were compared to ensure consistency and accuracy with the original content for referential adequacy (Lincoln & Guba, 2013). Second, in line with Olmos-Vega et al. (2022), the researcher engaged in reflexive practices to acknowledge how personal experiences and preconceptions about older women and IPH might influence the study. As described by Moustakas (1994), this was achieved through multiple rounds of reflection on preconceptions and biases.

## Findings

The findings of this study reveal that older women survivors of an attempted IPH encounter a complex interplay of physical, emotional, and social challenges that affect how they perceive and navigate their health situation, as represented by the following themes: (a) the ripple effect of the injury; (b) reduced access to formal and informal health-related support systems; and (c) limited financial resources for recovery needs.

### Theme 1: The ripple effect of the injury

The participants of this study reflected on the profound effect of attempted IPH on their physical health, including prolonged recovery time and exacerbation of pre-existing health conditions, as illustrated here:

The doctors said recovery is no picnic at my age… I’ve been hospitalized because of him before, but it never took this long; I spent weeks in the hospital and then in bed at home, couldn’t move at all… when I was younger, I never paid attention to being hurt physically because I was so hurt emotionally, but now my struggle has sort of changed; I’m devastated because of the pain and being dependent on others to do basic stuff for me… in some ways … I’m so concerned about managing my health, I can’t even feel anything else… (Adele, 69)

Through her long victimization history, Adele describes how aging has impacted her body’s resilience to injury, affecting her physical recovery over the years. Her experience as an older survivor brings out a unique shift in her perception of harm—in earlier years, she appeared more able to endure physical injury by focusing on emotional trauma. However, her struggles are no longer only psychological but profoundly physical, reflecting how the aging body, combined with past trauma, forces her to confront new levels of dependency and physical limitation. This loss of autonomy seems to affect her more deeply in older age, as dependency may symbolize not only a current limitation but also a more permanent loss. Her account suggests that her physical condition now dominates her emotional landscape, leaving little room for other feelings. Yet, despite these limitations, her persistence in managing her health reveals strength in the face of ongoing adversity.

While Adele pinpointed a new reality of navigating pain, Bella described how the attempted IPH has increased her struggle with some existing conditions:

Do you see I’m using a walker? This is new, since the hospital… the SOB tried to choke me, so what has that to do with my legs? … The age factor… when you’re old, it’s all connected somehow; you see, I have osteoporosis, and was unconscious in the hospital, and then regained consciousness but still couldn’t get up, and when I did stand up, my legs were very weak… you know, you think about women who survived against all odds, a lifetime of agony … who deserve to live freely now; there is no walker in this picture, right? It doesn’t add up… (Bella, 71)

Bella’s walker, which she associates with advanced age and dependency, is a powerful symbol of her changed reality. Her account conveys a sense of her body’s betrayal, as complications in her legs followed an attack on her upper body. Her reference to age reflects an understanding that older individuals’ health issues are interconnected, prolonging, and complicating recovery. She also conveys an expectation that old age would bring peace and autonomy, particularly after enduring prolonged hardship. She contrasts her envisioned survival, a strong woman free from limitations, with the reality of needing a walker, capturing the gap between her hoped-for independence and the sense of loss, as her anticipated freedom now seems unattainable. Nonetheless, in articulating her frustration and continuing to engage with care, Bella demonstrates the agency of someone still fighting for dignity and autonomy.

Beatrice adds:

Even if I had fully recovered (and I haven’t), I still get dizzy spells, now and again … I fell twice in the street, and at least three times around the house.… I fractured my hip and needed hospitalization again. You see, the murder, the attack, and the recovery; that was not the ending. It was the beginning of so many health issues. I honestly don’t know what caused me more agony, being knocked unconscious with him trying to choke me or all the pain since… (Beatrice, 66)

Beatrice describes how the trauma left her in a state of chronic vulnerability. Her frequent dizzy spells and repeated falls, leading to serious injuries, illustrate how her husband’s assault extended far beyond immediate harm. Her reflections emphasize that recovery marked the beginning of a series of ongoing health issues rather than a conclusion. The attempted homicide was not a singular event but a catalyst for ongoing medical challenges. Her account challenges the common assumption that survival marks the end of trauma and instead shows how its aftermath can be equally, if not more, debilitating.

### Theme 2: Reduced access to formal and informal Health-Related support systems

Most participants described having limited or disrupted support systems that either preceded the attempted IPH or were further eroded in its aftermath, leaving them to manage their new health challenges largely on their own:

I waited so long finally to be free of him, but now that’s happened, I don’t know what I was expecting. I’m alone all the time. I never drove; he didn’t want me to, and now it’s too late. If I run out of groceries, there’s no one to get them for me. I just make do with what’s left, and sometimes go without. My kids aren’t nearby, and we’re not very close. My parents are gone, my sister and I haven’t spoken in years, and the few connections I once had just slipped away. I don’t think that can change now… I can’t rely on anyone for a ride, can’t afford a taxi, so I end up skipping doctor’s appointments or missing medicine until I figure something out… And it’s not just the errands, it’s the little things, too. Since the attack, I can’t do much around the house without feeling exhausted. Cleaning wears me out, and it’s like I’m constantly reminded of what he took from me, piece by piece… (Dolores, 75)

After waiting for years to be freed from her husband’s violence, Dolores faces deep loneliness and disappointment with her new, helpless, isolated reality. The delicate webs spun around Dolores throughout her life were abruptly torn apart during the attempted IPH that removed her husband from her life. While Dolores’s perseverance enables her to manage day-to-day survival on her own, years in a violent relationship had stripped her of family ties, friendships, and even basic life skills such as driving. She perceives rebuilding lost connections and abilities as unattainable, feeling that change is no longer possible. Thus, she is on the verge of physical neglect and social detachment. Her sense of loss seems to encompass not only the attack itself but also the past and present life that might have been possible had she not been an IPV victim. Despite this situation, Dolores continues to manage her day-to-day survival on her own, reflecting a quiet perseverance in the face of abandonment.

While Dolores emphasizes the lack of a social support system, Tessa struggles to receive formal support:

I’m always at the doctor’s … They think I’m just a moaning old lady. They don’t know what to do with me, don’t see what I’ve been through, what I’ve survived. I guess I’m not that sick compared to other older folks, but I’m no longer the ordinary woman running from her husband, with all her strength, … I’ve been shuffled between specialists, and no one seems to want responsibility for my care… They treat each injury separately, and when I try to get some follow-up from the same doctor, they refuse to set an appointment… When I was a young mother, I had no problem getting doctor’s appointments; now, I feel no one cares if I make it through another day… When I try to explain the pain or my struggles just getting around, they brush it off, like it’s just part of aging and I should deal with it, and not the result of everything he did to me… (Tessa, 73)

Tessa’s account reflects how broader systemic shortcomings in healthcare, such as fragmented services and lack of care continuity, are experienced in uniquely harmful ways by older survivors of attempted IPH. While many patients may face bureaucratic barriers, Tessa’s struggles are intensified by age-related dismissal, as providers appear to minimize her symptoms and overlook the traumatic origins of her health problems.

The apparent lack of attention to her history of violence may contribute to care that feels impersonal, inconsistent, and disconnected from her lived experience. This could lead to feelings of being invalidated or deprioritized, especially when contrasted with earlier life stages during which she recalls receiving more responsive care. Her account suggests that the combined effects of aging and survivorship may create distinct barriers to healthcare that are not fully captured by broader critiques of the system.

Similarly, Ella feels unsupported in accessing health resources, linking this struggle to her difficulty navigating modern health systems’ technological demands:

Getting help for myself is an ongoing battle, like I haven’t already had my share of fighting… I’ve been chasing the physical therapy unit for months, trying to get an appointment. I don’t know how to use those apps or websites they tell me about… I’ve called so many times, and last week, even took the bus to the doctor’s clinic and sat there for hours, hoping someone would help me. Everyone was very polite, of course, no one would be rude to an old lady, but no one took me seriously either. They just said I didn’t have an appointment and sent me home. That was it. It’s not just about getting one appointment; it’s about keeping track of all these different treatments and therapies; I can’t manage everything on my own. When I ask for help, they hand me pamphlets or suggest programs that don’t even apply to me… (Ella, 62)

Ella describes seeking medical help as an “ongoing battle,” directly mirroring her past struggle to survive. For her, accessing care continues the cycle of effort and resilience once required to endure the violent relationship and attempted IPH. This battle also encompasses the technological challenges of navigating modern-day health systems. Lacking digital literacy, Ella depends on phone calls and physical visits that mostly lead to nothing. The case of her waiting for hours at the clinic and then being dismissed reflects the staff’s impersonal attitude, despite her obvious effort and need. Ella’s remark highlights covert ageism—although treated politely, her concerns are dismissed and her lack of digital access is ignored, leaving her without adequate care. Struggling to manage fragmented treatments, Ella faces a healthcare system that places the burden on her, a challenge she cannot meet without support. Yet even as she encounters repeated setbacks, her continued efforts to seek care, follow-up on referrals, and advocate for herself despite these obstacles reveal a tenacious will to recover and be heard.

### Theme 3: Limited financial resources and recovery needs

Financial precarity emerged as a third, distinct barrier to recovery. Participants described three intertwined pressures: the out-of-pocket cost of everyday treatments, the ongoing legal fees required to keep an assailant behind bars, and the need to return to work in later life to fund essential care. Years of economic control had already eroded their savings, and these new expenses locked survivors into a continuing cycle of strain and unmet health needs, which they navigated with determination despite severe constraints, as the following accounts illustrate.

What hurts the most is knowing that if I had money, I could get better care. People think older women are just supposed to have everything sorted out by now, that I don’t have kids to take care of, that I must be well off, but living with my husband left me with nothing. When I was younger, I worked here and there, but he never let me have a real career… I never had the chance to save or build anything for myself… never could put money aside for a rainy day… my back, my neck, my legs, everything hurts—and my doctor tells me I need therapy, special treatments, medications. But none of it is covered fully, and I can’t afford it. I’m on a fixed income, barely getting by with my pension… (Rachel, 69)

Despite the significant health needs resulting from Rachel’s attempted IPH injuries and the toll of aging, she cannot afford the recommended therapies and medications. Her fixed income and modest pension leave her feeling trapped, with no way to bridge the gap between her needs and available care. Her account highlights the long-term effects of financial control often present in abusive relationships. Her inability to build financial security has left her particularly vulnerable in older age, when stable resources are critical for addressing health concerns. Her husband’s financial control during their marriage not only stripped her of economic independence but also limited her ability to prepare for the future, leaving her to face her current challenges without the means to address them adequately.

Where Rachel highlights the burden of routine healthcare expenses, Stella’s account shifts the focus to the crippling legal costs that further drain limited resources:

Every penny goes to fighting to keep him in prison. It’s been years; and every time there’s a hearing or an appeal, I have to hire another lawyer or pay for more paperwork. I can’t sleep at night, thinking about what would happen if he got out… I don’t have anything left for myself, for my health, for the therapy they say I need. The doctors suggest things, and I just nod because I know I can’t pay for them. Everything I have goes on rent and food, let al.ne this constant legal battle… It’s like I’m still trapped, even with him behind bars. He’s taken everything from me—my health, my money, my peace of mind… (Stella, 60)

Stella’s narrative illustrates how her ongoing legal battles to keep her attacker incarcerated deepen her vulnerability, trapping her in a cycle of financial and emotional strain that undermines recovery. This financial burden directly affects her ability to prioritize her health. Her account conveys painful resignation—Doctors’ recommendations for medical treatments and therapies feel meaningless when she knows she cannot afford them. Her reaction reflects an awareness of the gap between her needs and what is attainable, reinforcing her sense of helplessness. Stella’s persistent fear, coupled with untreated health issues, ties her present to the attempted IPH trauma, leaving her feeling perpetually under threat, even with her attacker behind bars.

While Stella’s finances were consumed by legal fees, Becky responded to her own shortfall by re-entering the workforce at age 70:

Sometimes I wonder if it’s worth it; working myself to the bone just for a bit of relief. But then I think, at least this way, I’m fighting for something. I never saw myself going back to work at 70 but here I am… the decision to find a job wasn’t hard—after the relief of hydrotherapy and I used up all my free sessions, it was clear I’d do whatever I can to keep getting this treatment, even if it meant turning my life around… (Becky, 70)

Becky’s decision to return to work at 70 reflects her struggle to adapt to a reality that defies societal norms for older adults. This unexpected choice highlights her severe health challenges, particularly her reliance on hydrotherapy to manage pain and aid recovery. The limit on free sessions leaves her with no option but to fund ongoing treatment herself. For Becky, work is a necessity rather than a choice, underscoring how structural gaps in healthcare access burden older survivors with limited financial resources. Her narrative reveals an internal conflict: The physical toll of working at her age exacerbates exhaustion and pain, making her question the trade-off. At the same time, she views work as a form of resistance and agency, demonstrating her determination to reclaim a sense of control over her health and her life.

Collectively, the women’s accounts demonstrate how medical costs, legal fees, and late-life employment converge to intensify the health inequities imposed by financial precarity.

## Discussion and implications

The findings indicate that older women survivors of IPH endure multiple health burdens, including persistent pain, mobility challenges, and other lingering effects that exacerbate preexisting conditions (Theme 1). They experience limited access to formal and informal support, such as medical follow-up and social networks, which are critical for recovery (Theme 2). Constrained financial resources further restrict their ability to secure ongoing care, increasing vulnerability and limiting their path to better health (Theme 3). These patterns illustrate a cumulative burden that has accrued over decades of abuse and structural disadvantage. Long-term exposure to IPV not only layered physical and psychological harm but also entrenched financial dependency and social isolation conditions that limited women’s power to negotiate care and shaped how they experienced the attempted IPH in late life and their subsequent recovery.

Some of these findings are consistent with research concerning younger attempted IPH survivors; for example, Evans (2018) and Vella et al. (2017) reported attempted IPH survivors’ need for extended medical attention following the violent incident, including surgery and physical therapy; however, barriers to obtaining this help were not described. Moreover, while some studies reported survivors’ tendency not to seek the support of formal caregivers (including health professionals), this seemed to be a part of younger survivors’ choice to distance themselves from such resources (Santucci, 2021; Vásquez et al., 2025) rather than due to systemic barriers such as lack of transportation and technical illiteracy, as exemplified in this study. In other words, the participants in this study appeared to desire help that was beyond their reach. In addition, although financial hardship frequently emerges as a challenge for IPH survivors, findings of previous studies showed that it did not significantly influence their ability to navigate healthcare. Farr (2002) found general satisfaction with medical care, among participants, and although some mentioned financial difficulties following the attempted IPH, these challenges did not seem to impede access to necessary services. Similarly, Evans (2018) reported post-IPH financial obstacles but did not connect them to barriers to obtaining healthcare.

In addition to studies on older IPH survivors, studies concerning older women who survived severe physical IPV offer a valuable context for understanding the current findings. For example, some studies show how untreated injuries and health issues from earlier IPV intensify with age, compounding age-related challenges such as bone fragility or reduced mobility, thereby strengthening this study’s finding regarding the ripple effect of the attempted IPH and its consequences for ongoing health issues (Cannell et al., 2015; Pathak et al., 2019). In addition, studies have highlighted issues hindering older survivors’ access to support, exemplifying how healthcare providers often fail to screen for IPV among older women (Zink et al., 2004), or misattribute its impacts to normal aging (Eisikovits & Band-Winterstein, 2015). These barriers to healthcare support appear to differ from those found in this study, where participants’ health issues were recognized and treated; however, actual receipt of treatment remained challenging due to logistical hurdles or a fragmented system with excessive bureaucracy. Finally, some IPV studies identified the connection between prolonged financial dependence on abusers, current financial challenges, and difficulty acquiring health care (Pathak et al., 2019). The findings of this study further illuminated this triangular connection, highlighting how “normal” aging can be complicated by financial challenges.

The findings of this study can be viewed through an intersectional lens, highlighting those overlapping systems of oppression or privilege that shape individuals’ experiences. One particularly relevant concept in this framework is “embodied intersectionality,” which emphasizes that these overlapping forces are not merely social or psychological but also manifest in the body. This approach considers how people’s physical health, body image, stress responses, and overall bodily experiences are directly shaped by the intersecting social identities they inhabit. As Mirza (2013) suggests, embodied intersectionality can be viewed as a practical way of mapping the effects of intersectionality on the body, thereby operationalizing the theory. In this study, in Theme 1 (the ripple effect of the injury), for example, Adele’s account highlights how her victimization history intersects with aging, resulting in intensified physical pain and new dependencies that reshape her daily life. In Theme 2 (reduced access to formal and informal support systems), Tessa’s experience of being dismissed by healthcare professionals shows how ageism and the aftermath of violence combine to restrict her access to appropriate care, ultimately exacerbating her health challenges. These examples underscore how survivors’ bodies are the sites where various social and structural forces collide, shaping their long-term recovery and well-being.

Another intersectional theory concept that can help shed light on the findings of this study is intersectional invisibility. According to Purdie-Vaughns and Eibach (2008), when individuals with multiple subordinate-group identities (e.g., older women, survivors of IPH) do not fit their respective identity group prototypes (e.g., either older women or IPH survivors), they may experience intersectional invisibility. This refers to a general failure to fully recognize such individuals as members of their constituent groups, leading to marginalization, misrepresentation, and a unique set of social disadvantages and advantages compared to prototypical members of those groups. In this study, Theme 2 brings forth Tessa’s account, which illustrates how her unique position as an older woman survivor of IPH renders her “invisible” to medical providers. She does not match the prototypical profile of a younger IPV survivor—perceived as healthy but vulnerable, nor does she fit the stereotypical image of an older patient with solely age-related ailments. Consequently, her chronic pain and history of violence are dismissed as natural consequences of aging, leading to fragmented and inconsistent care. Similarly, in Theme 3, Rachel’s narrative indicates how she, too, falls outside expected norms. Contrary to assumptions that older women are financially stable, her years of economic abuse and lack of career opportunities left her unprepared for both routine and specialized medical expenses. Thus, neither archetype—neither older adults with secure retirement, nor typical IPV survivors—encompasses her struggles, resulting in intersectional invisibility and marginalization of her needs.

Finally, the concept of intersecting power relations shows how multiple levels of oppression (from personal to structural) converge and shape experiences of violence. While intersectionality broadly addresses overlapping social categories, this concept focuses on the power imbalances within those intersections. Hill Collins and Bilge (2020) note its relevance to gendered violence, which both enforces and stems from systems such as racism, heteropatriarchy, and nationalism, leading women from diverse backgrounds to experience violence in distinct ways. In the second and third themes, the participants describe struggles to secure adequate medical care, receive attention from healthcare providers, and follow treatment plans to improve their health. However, systemic barriers, for example, limited digital literacy in navigating healthcare technology and a lack of reliable transportation, hinder these efforts. Thus, the intersection of aging-related challenges and a personal history of IPV places these individuals in a vulnerable, underprivileged position within the healthcare system’s relational hierarchy that ultimately leaves them overlooked and underserved.

### Conclusion

This study shows that older women who survive an attempted IPH must navigate interconnected physical, emotional, and socioeconomic challenges that stretch well beyond the violent event itself. A lifetime of gendered, age-related, and economic power imbalances compounds the severity of the attack, narrowing the pathways to recovery and producing persistent injuries, limited support, and financial strain. Recognizing this cumulative burden is important because health inequities, constrained resources, and social marginalization converge to intensify survivors’ late-life recovery needs.

### Limitations and recommendations for further study

While the findings offer rich insights, they are not intended to be universally representative due to the study’s small sample size and qualitative approach. Future studies aiming for more generalizable results may incorporate larger sample sizes across diverse cultural contexts.

In addition, all participants in this study were women who survived attempted IPH perpetrated by male partners. Future research should examine the experiences of individuals across a broader range of gender identities and sexual orientations to capture the diversity of intimate partner violence contexts. Furthermore, participants’ health experiences may be shaped by factors such as age, duration of pre-IPH abuse, injury severity, and overall health status. Future research with broader samples could deepen understanding of these variations and survivors’ experiences.

In addition, recruiting individuals who had publicly shared their stories helped navigate ethical challenges inherent in studying attempted IPH survivors but may have excluded women who did not disclose their experiences. Future research should employ broader methods to include such voices. In this context, while this study draws on intersectionality as a conceptual framework, it focuses primarily on the intersection of age and victimization and does not directly examine race or social class, key dimensions highlighted in related work on older IPV/IPH survivors (e.g., Harper, 2017; Posey, 2024). Future research that incorporates additional identity dimensions could offer a more comprehensive picture of how multiple forms of marginalization shape older survivors’ experiences.

While the themes identified in this study emphasize the structural and embodied challenges faced by older IPH survivors, the narratives also reflect moments of resilience, agency, and resistance (Roberto, 2015). Future research could explore how these strengths are enacted and sustained in long-term recovery.

Finally, as participants’ perceptions may change as they age and their health situation evolves, longitudinal studies are needed to explore these dynamic processes.

### Practical implications

The findings emphasize the need to apply an age-sensitive, trauma-informed, and intersectional lens to IPV and IPH frameworks, which have traditionally focused on the experiences of younger survivors. This study illustrates how the intersection of aging, survivorship, and structural neglect produces unique health burdens that require more than generic post-violence care. Older survivors of attempted IPH experience compounded vulnerabilities, including chronic pain, mobility limitations, technological exclusion, financial precarity, and social isolation, which converge to hinder recovery and often remain invisible in existing policy and practice frameworks.

Policymakers should consider expanding legislation and funding mechanisms to support long-term, integrative care models that account for the specific needs of older survivors. For example, subsidized access to rehabilitative therapies, trauma-informed counseling, and home-based care services can address persistent injuries and mobility-related barriers. In addition, policies that guarantee transportation support for medical and legal appointments or provide alternatives to digital-only health access are essential for survivors who are physically or technologically constrained. Finally, legal aid and survivor-centered justice initiatives must be extended to account for the ongoing legal struggles that many older women face, which often compete with their ability to manage basic health needs. These reforms would help dismantle systemic barriers that disproportionately affect older survivors and reframe recovery as a long-term and structural commitment rather than a short-term intervention.

In practice, healthcare providers play a crucial role in translating these principles into meaningful care. Age-aware and trauma-informed clinical practices should be embedded into routine care settings. This may include offering longer or more flexible appointments to accommodate cognitive fatigue or chronic pain, simplifying scheduling systems, and ensuring accessible communication verbally, in writing, and digitally. Providers should also adopt a coordinated approach across disciplines, linking primary care, rehabilitation, mental health services, and community-based supports to avoid fragmented treatment that places the burden of navigation on survivors. In addition, routine assessment tools and screening practices must be adapted to capture the less visible health consequences of violence in later life, which may be misattributed to aging or overlooked altogether. These measures can help address not only the physical injuries but also the psychosocial toll of surviving an attempted IPH, particularly in cases where survivors, like those in this study, express a clear desire for support that remains out of reach.

Together, these policy and practice approaches move beyond crisis response and toward sustainable and survivor-centered models that acknowledge the intersecting forces shaping older women’s post-violence health.

## Supplementary Material

gnaf287_Supplementary_Data

## Data Availability

The author confirms that the study has not been preregistered. The data underlying this article cannot be shared due to ethical restrictions.

## References

[gnaf287-B1] Abrunhosa C. , de Castro Rodrigues A., Cruz A. R., Gonçalves R. A., Cunha O. (2021). Crimes against women: From violence to homicide. Journal of Interpersonal Violence, 36, NP12973–NP12996. 10.1177/0886260520905547 32046602

[gnaf287-B2] Alessandrino F. , Keraliya A., Lebovic J., Mitchell Dyer G. S., Harris M. B., Tornetta P., Boland G. W. L., Seltzer S. E., Khurana B. (2020). Intimate partner violence: A primer for radiologists to make the “invisible” visible. Radiographics: a Review Publication of the Radiological Society of North America, Inc, 40, 2080–2097. 10.1148/rg.2020200010 33006922

[gnaf287-B3] Avieli H. (2025a). The emotional aftermath of surviving an attempted intimate partner homicide. Qualitative Health Research, 35, 44–55. 10.1177/10497323241245643 PMC11626850 39091209

[gnaf287-B4] Avieli H. (2025b). The social response to survivors of attempted intimate partner homicide. Violence against Women, 10778012251319318. 10.1177/10778012251319318 PMC12913690 39957132

[gnaf287-B5] Avieli H. , Band-Winterstein T. (2024). The multiple punishment of being an older adult coping with health problems in prison. The Gerontologist, 64, gnad030. 10.1093/geront/gnad030 36943327

[gnaf287-B6] Azevedo A. K. S. , Dutra E. M., do S. (2015). There is no you without me: Stories of surviving women of a murder attempt. Revista Subjetividades, 15, 201–213.

[gnaf287-B8] Benbow S. M. , Bhattacharyya S., Kingston P. (2019). Older adults and violence: An analysis of domestic homicide reviews in England involving adults over 60 years of age. Ageing and Society, 39, 1097–1121. 10.1017/S0144686X17001386

[gnaf287-B9] Best D. , Van Wersch A., Carthy N. (2021). “It’s like drowning and you can’t get out”: The influence of intimate partner violence on women with chronic low back pain. The European Journal of Counselling Psychology, 9, 7–16. 10.46853/001c.22008

[gnaf287-B10] Bourget D. , Gagné P., Whitehurst L. (2010). Domestic homicide and homicide-suicide: The older offender. The Journal of the American Academy of Psychiatry and the Law, 38, 305–311. 20852214

[gnaf287-B11] Bows H. (2019). Domestic homicide of older people (2010–15): A comparative analysis of intimate-partner homicide and parricide cases in the UK. The British Journal of Social Work, 49, 1234–1253. 10.1093/bjsw/bcy108

[gnaf287-B12] Breitman N. , Shackelford T. K., Block C. R. (2004). Couple age discrepancy and risk of intimate partner homicide. Violence and Victims, 19, 321–342. 10.1891/vivi.19.3.321.65764 15631284

[gnaf287-B13] Cannell M. B. , Weitlauf J. C., Garcia L., Andresen E. M., Margolis K. L., Manini T. M. (2015). Cross-sectional and longitudinal risk of physical impairment in a cohort of postmenopausal women who experience physical and verbal abuse. BMC Women’s Health, 15, 98. 10.1186/s12905-015-0258-2 PMC4641397 26554450

[gnaf287-B14] Cantor E. , Salas R., Torres R. (2022). Femicide and attempted femicide before and during the COVID-19 pandemic in Chile. International Journal of Environmental Research and Public Health, 19, 8012. 10.3390/ijerph19138012 PMC9265640 35805670

[gnaf287-B17] Crenshaw K. (1989). Demarginalizing the intersection of race and sex: A black feminist critique of antidiscrimination doctrine, feminist theory and antiracist politics. The University of Chicago Legal Forum, 140, 139–167.

[gnaf287-B18] Cullen P. , Dawson M., Price J., Rowlands J. (2021). Intersectionality and invisible victims: Reflections on data challenges and vicarious trauma in femicide, family and intimate partner homicide research. Journal of Family Violence, 36, 619–628. 10.1007/s10896-020-00243-4 PMC7854328 33551548

[gnaf287-B19] Eisikovits Z. , Band-Winterstein T. (2015). Dimensions of suffering among old and young battered women. Journal of Family Violence, 30, 49–62. 10.1007/s10896-014-9655-9

[gnaf287-B20] Evans D. P. , Williams N. S. D., Wilkins J. D., Chiang E. D., Manders O. C., Vertamatti M. A. F. (2018). “He said he was going to kill me”: Case studies of attempted intimate femicide in São Paulo, Brazil. Journal of Comparative Social Work, 13, 57–80. 10.31265/jcsw.v13i1.159

[gnaf287-B21] Farr K. A. (2002). Battered women who were “being killed and survived it”: Straight talk from survivors. Violence and Victims, 17, 267–281. 10.1891/0886-6708.17.3.267 12102053

[gnaf287-B22] Fay D. L. , Hicklin Fryar A., Meier K. J., Wilkins V. (2021). Intersectionality and equity: Dynamic bureaucratic representation in higher education. Public Administration, 99, 335–352. 10.1111/padm.12689

[gnaf287-B23] Gaber S. N. , Mattsson E., Klarare A., Dawes J., Rapaport P,.;Women’s Advisory Board for Inclusion Health. (2025). An intersectional perspective on digital health: Longitudinal narratives and observations with older and middle-aged women experiencing homelessness. Gerontologist, 65, gnaf021. 10.1093/geront/gnaf021 PMC11973565 39869439

[gnaf287-B24] Graham L. M. , Macy R. J., Rizo C. F., Martin S. L. (2022). Explanatory theories of intimate partner homicide perpetration: A systematic review. Trauma, Violence & Abuse, 23, 408–427. 10.1177/1524838020953800 32909896

[gnaf287-B25] Gray A. C. , Kafonek K., Parker K. F. (2024). Firearms, policy, and intimate partner homicide: A structural and disaggregated examination of Black, Latina, and White female victimization. Criminology, 62, 276–299. 10.1111/1745-9125.12372

[gnaf287-B26] Harden J. , Du J., Spencer C. M., Stith S. M. (2019). Examining attempted and completed intimate partner homicide: A qualitative synthesis. Violence and Victims, 34, 869–888. 10.1891/0886-6708.VV-D-18-00128 31836641

[gnaf287-B27] Harper S. B. (2017). No way out: Severely abused Latina women, patriarchal terrorism, and self-help homicide. Feminist Criminology, 12, 224–247. 10.1177/1557085116680743

[gnaf287-B28] Harper S. B. (2022). “I’m just like, you know what, it’s now or never”: Exploring how women of color experiencing severe abuse and homicide risk journey toward formal help-seeking. Journal of Interpersonal Violence, 37, NP13729–NP13765. 10.1177/08862605211005150 33849299

[gnaf287-B30] Hill Collins P. (2009). Black feminist thought: Knowledge, consciousness, and the politics of empowerment (2nd ed.). Routledge.

[gnaf287-B31] Hill Collins P. , Bilge S. (2020). Intersectionality (2nd ed.). Polity Press.

[gnaf287-B32] Kavanaugh K. , Ayres L. (1998). “Not as bad as it could have been”: Assessing and mitigating harm during research interviews on sensitive topics. Research in Nursing & Health, 21, 91–97. 10.1002/(sici)1098-240x(199802)21:1<91::aid-nur10>3.0.co; 2-c 10.1002/(sici)1098-240x(199802)21:1<91::aid-nur10>3.0.co;2-c 9472241

[gnaf287-B16] Kennedy B. , Bugeja L., Olivier J., Koppel S., Dwyer J., Ibrahim J. (2023). A population-based cross-sectional study examining homicides among community-dwelling older adults in Victoria, Australia: A study protocol. PloS One, 18, e0292837. 10.1371/journal.pone.0292837 PMC10575534 37831701

[gnaf287-B33] Kivisto A. J. (2015). Male perpetrators of intimate partner homicide: A review and proposed typology. The Journal of the American Academy of Psychiatry and the Law, 43, 300–312. 26438808

[gnaf287-B34] Liamputtong P. (2007). Researching the vulnerable: A guide to sensitive research methods. Sage.

[gnaf287-B35] Lincoln Y. S. , Guba E. G. (2013). The constructivist credo. Left Coast Press, Inc.

[gnaf287-B36] MacPherson M. , Reif K., Titterness A., MacQuarrie B. (2020). Older women and domestic homicide. In Preventing domestic homicides. (pp. 15–37). Elsevier. 10.1016/B978-0-12-819463-8.00002-2

[gnaf287-B37] Mirza H. S. (2013). ‘A second skin’: Embodied intersectionality, transnationalism and narratives of identity and belonging among Muslim women in Britain. Women’s Studies International Forum, 36, 5–15. 10.1016/j.wsif.2012.10.012

[gnaf287-B38] Moustakas C. (1994). Phenomenological research methods. Sage Publications, Inc. 10.4135/9781412995658

[gnaf287-B39] Mouton C. P. (2003). Intimate partner violence and health status among older women. Violence Against Women, 9, 1465–1477. 10.1177/1077801203259232

[gnaf287-B40] Nicolaidis C. , Curry M. A., Ulrich Y., Sharps P., McFarlane J., Campbell D., Gary F., Laughon K., Glass N., Campbell J. (2003). Could we have known? A qualitative analysis of data from women who survived an attempted homicide by an intimate partner. Journal of General Internal Medicine, 18, 788–794. 10.1046/j.1525-1497.2003.21202.x. PMC1494930 14521640

[gnaf287-B41] Olmos-Vega F. M. , Stalmeijer R. E., Varpio L., Kahlke R. (2022). A practical guide to reflexivity in qualitative research: AMEE Guide No. 149. Medical Teacher, 45, 1–251. 10.1080/0142159X.2022.2057287 35389310

[gnaf287-B42] Pathak N. , Dhairyawan R., Tariq S. (2019). The experience of intimate partner violence among older women: A narrative review. Maturitas, 121, 63–75. 10.1016/j.maturitas.2018.12.011 PMC6546119 30704567

[gnaf287-B43] Patton M. Q. (2015). Qualitative research & evaluation methods: Integrating theory and practice. (4th ed.) Sage Publications, Inc.

[gnaf287-B44] Pietkiewicz I. , Smith J. (2014). A practical guide to using Interpretative Phenomenological Analysis in qualitative research psychology. Czasopismo Psychologiczne Psychological Journal, 20, 1, 7–14. 10.14691/CPPJ.20.1.7

[gnaf287-B45] Posey B. M. (2024). Black femicides matter: Conceptualizing the killings of black girls and women as structural and cultural violence. Homicide Studies, 28, 313–340. 10.1177/ 10887679231209227

[gnaf287-B46] Purdie-Vaughns V. , Eibach R. P. (2008). Intersectional invisibility: The distinctive advantages and disadvantages of multiple subordinate-group identities. Sex Roles, 59, 377–391. 10.1007/s11199-008-9424-4

[gnaf287-B47] Rahman M. (2008). Theorising intersectionality: Identities, equality, and ontology. In Intersectionality and beyond (pp. 368–389). Routledge-Cavendish.

[gnaf287-B48] Roberto K. A. , McCann B. R., Brossoie N. (2013). Intimate partner violence in late life: An analysis of national news reports. Journal of Elder Abuse & Neglect, 25, 230–241. 10.1080/08946566.2012.751825 PMC3641680 23627429

[gnaf287-B49] Roberto K. A. (2015). November) Resilience in the presence of adversity: Overcoming partner violence in late life. Gerontologist, 55(Suppl_2), 367. 10.1093/geront/gnv608.02

[gnaf287-B50] Salari S. (2007). Patterns of intimate partner homicide suicide in later life: Strategies for prevention. Clinical Interventions in Aging, 2, 441–452. https://www.semanticscholar.org/paper/Patterns-of-intimate-partner-homicide-suicide-in-Salari/7712d7885ffe7bd3f05d224687ee92c2ff166f74 PMC2685270 18044194

[gnaf287-B51] Salari S. , Maxwell C. D. (2016). Lethal intimate partner violence in later life: Understanding measurements, strengths, and limitations of research. Journal of Elder Abuse & Neglect, 28, 235–262. 10.1080/08946566.2016.1247402 27732523

[gnaf287-B52] Salari S. , Sillito C. L. (2016). Intimate partner homicide–suicide: Perpetrator primary intent across young, middle, and elder adult age categories. Aggression and Violent Behavior, 26, 26–34. 10.1016/j.avb.2015.11.004

[gnaf287-B53] Santucci D. M. P. (2021). *Help-seeking behaviors of survivors of intimate partner femicide attacks* [Doctoral dissertation, Walden University]. https://www.proquest.com/openview/0796ceff6c804f02a939c80870199619/1? pq-origsite=gscholar&cbl=18750&diss=y

[gnaf287-B54] Saxe A. (2017). The theory of intersectionality: A new lens for understanding the barriers faced by autistic women. Canadian Journal of Disability Studies, 6, 153–178. 10.15353/cjds.v6i4.386

[gnaf287-B55] Smith J. A. , Flowers P., Larkin M. (2009). Interpretative phenomenological analysis: Theory, method and research. Sage.

[gnaf287-B56] Smith J. A. , Nizza I. E. (2022). Essentials of interpretative phenomenological analysis. American Psychological Association. 10.1037/0000259-000

[gnaf287-B57] Sosa L. (2023). Femicide and intersectionality. In Dawson M., Vega S. M. (Eds.), The Routledge international handbook on femicide and feminicide. (pp. 50–59). Routledge. 10.4324/9781003202332-7

[gnaf287-B58] Stubbs A. , Szoeke C. (2022). The effect of intimate partner violence on the physical health and health-related behaviors of women: A systematic review of the literature. Trauma, Violence, & Abuse, 23, 1157–1172. 10.1177/1524838020985541 33541243

[gnaf287-B59] Sun S. , Crooks N., Kemnitz R., Westergaard R. P. (2018). Re-entry experiences of Black men living with HIV/AIDS after release from prison: Intersectionality and implications for care. Social Science & Medicine (1982), 211, 78–86. 10.1016/j.socscimed.2018.06.003 PMC6364300 29913303

[gnaf287-B60] Thomas R. , Dyer G. S. M., Tornetta Iii P., Park H., Gujrathi R., Gosangi B., Lebovic J., Hassan N., Seltzer S. E., Rexrode K. M., Boland G. W., Harris M. B., Khurana B. (2021). Upper extremity injuries in the victims of intimate partner violence. European Radiology, 31, 5713–5720. 10.1007/s00330-020-07672-1 PMC7812562 33459857

[gnaf287-B61] Thomas K. A. , Joshi M., Sorenson S. B. (2014). “Do you know what it feels like to drown?”: Strangulation as coercive control in intimate relationships. Psychology of Women Quarterly, 38, 124–137. 10.1177/0361684313488354

[gnaf287-B62] United Nations Office on Drugs and Crime. (2019). *Global study on homicide.* Available from: https://www. unodc. org/docum ents/data- and-analysis/gsh/Booklet2.pdf

[gnaf287-B63] UNODC, & UN Women. (2024). Femicides in 2023: Global estimates of intimate partner/family member femicides. United Nations.

[gnaf287-B64] Vásquez L. , Kim C., Rajah V. (2025). Intimate partner violence in El Salvador: A relationship between femicide attempts and barriers to help-seeking. Violence against Women, 31, 750–766. 10.1177/10778012231222489 38166483

[gnaf287-B65] Vatnar S. K. B. , Bjørkly S. (2013). Lethal intimate partner violence: An interactional perspective on women’s perceptions of lethal incidents. Violence and Victims, 28, 772–789. 10.1891/0886-6708.VV-D-12-00062 24364122

[gnaf287-B66] Vella S. A. , Miller M. M., Lambert J. E., Morgan M. L. (2017). “I felt close to death”: A phenomenological study of female strangulation survivors of intimate terrorism. Journal of Feminist Family Therapy, 29, 171–188. 10.1080/08952833.2017.1370572

[gnaf287-B67] Weil S. (2016). Failed femicides among migrant survivors. Qualitative Sociology Review, 12, 6–21. 10.18778/1733-8077.12.4.01

[gnaf287-B68] Weil S. , Keshet N. S. (2021). Female geronticide: The case of Israel. Journal of Gender Studies, 30, 39–51. 10.1080/09589236.2020.1809361

[gnaf287-B71] Wuest J. , Ford-Gilboe M., Merritt-Gray M., Varcoe C., Lent B., Wilk P., Campbell J. (2009). Abuse-related injury and symptoms of posttraumatic stress disorder as mechanisms of chronic pain in ­survivors of intimate partner violence. Pain Medicine (Malden, Mass.), 10, 739–747. 10.1111/j.1526- 4637. 2009.00624.x 10.1111/j.1526-4637.2009.00624.x 19453953

[gnaf287-B72] Wuest J. , Ford-Gilboe M., Merritt-Gray M., Wilk P., Campbell J. C., Lent B., Varcoe C., Smye V. (2010). Pathways of chronic pain in survivors of intimate partner violence. Journal of Women’s Health (2002), 19, 1665–1674. 10.1089/jwh.2009.1856 PMC3120089 20718626

[gnaf287-B74] Zink T. , Jeffrey Jacobson C., Regan S., Pabst S. (2004). Hidden victims: The healthcare needs and experiences of older women in abusive relationships. Journal of Women’s Health (2002), 13, 898–908. 10.1089/jwh.2004.13.898 15671705

