# Supplementary material for: Health challenges of older women following attempted intimate partner homicide
Source: Gerontologist. 2025 Dec 8;66(3):gnaf287. doi: 10.1093/geront/gnaf287 (PMC13604828; doi:10.1093/geront/gnaf287)
Supplement: Supplementary file 1 [file gnaf287_supplementary_data.docx]

**Appendix A – Participants**’ **Sociodemographic information**

The participant’s data is presented in an aggregated format to avoid identifying individuals, particularly in small samples, as recommended by Morse and Coulehan (2015).

All the participants in the current study (*N=9*) were women between the ages of 60 and 75 (*M=68.3*). They had all survived an attempted homicide at the hands of a partner (eight participants were married, and one had been attacked by her boyfriend). All the participants were attacked when over the age of 60, and the time that had elapsed between the attack and the interviews ranged from several months to 4 years.

All participants had spent time in the hospital following these attacks (*M=16 days*). The participants’ injuries resulted from gunshots, choking, stabbing, being thrown against the wall, having their heads pounded against the floor, and being hit with a heavy object. Five of the participants were aware of having a prior medical condition (one had recovered from cancer and the other four had osteoporosis, arthritis, hypertension, heart disease, and diabetes).

While all participants identified as white, they came from a range of ethnic and cultural backgrounds, including individuals from both majority and minority populations within their national context.

All the participants had children; however, three of them were not in touch with them. Most of the women were retired; one had returned to work after retirement, and two were still working occasionally in temporary jobs. Among the perpetrators, one had desisted, and the rest were incarcerated. At the time of the interviews, four participants were involved in legal proceedings concerning the attack. Six participants resided alone, and three lived with one of their adult children.

Morse, J. M., & Coulehan, J. (2015). Maintaining confidentiality in qualitative

publications. *Qualitative Health Research, 25(2)*, 151–152.

**Appendix B: Interview guide**

**Category 1 – The attempted IPH and its immediate health consequences**

1. What do you remember from the hours and days following the violent incident?
2. How did you feel physically and emotionally while still in the hospital? (if applicable)
3. What were the short‑term consequences of the violent attack for you?
4. Can you describe the injuries you sustained and how they affected you at the time?
5. How did you experience pain or discomfort during those early days?
6. In what ways, if any, did your age influence your immediate recovery?
7. What stands out for you about your first weeks at home after the attack?
8. How did you manage everyday tasks (e.g., dressing, bathing, cooking) right after the incident?
9. Did the attack aggravate any health conditions you already had? If so, how?
10. How did your need for help or dependence on others feel in those first weeks?
11. Were there moments when you realised recovery might take longer than you expected?
12. How did the medical environment (in the hospital - staff, ward, equipment) shape your sense of safety or vulnerability?
13. What emotions surfaced most strongly as you began to understand what had happened?
14. Looking back, is there anything about that immediate period you wish others had understood better?

**Category 2 – The experience of receiving medical care following the attack**

1. Can you describe the kind of medical care you require now and how you obtain it?
2. How do you feel about this ongoing healthcare?
3. What has been helpful—or unhelpful—in your interactions with healthcare professionals?
4. Have you faced any barriers (practical, emotional, financial) in accessing care?
5. How easy or difficult is it for you to schedule and attend follow‑up appointments?
6. In what ways do you feel your age affects how medical staff treat or listen to you?
7. Can you walk me through a recent experience of trying to get the care you needed?
8. How do you keep track of medications, therapies, or specialist visits?
9. Have you ever chosen not to seek care? What influenced that decision?
10. How does technology (e.g., online forms or apps) affect your ability to receive care?
11. What role, if any, do family or friends play in helping you manage your healthcare?
12. Do you feel your current care addresses the long‑term effects of the attack? Why or why not?
13. Have you experienced being dismissed or not taken seriously when explaining your pain or needs?
14. If you could change one thing about the healthcare you receive now, what would it be?
15. Can you recall a moment when you felt particularly supported—or unsupported—by the health system?

**Category 3 – A retrospective view of how the attempted IPH has impacted health**

1. What were the long‑term consequences of the violent attack for your health?
2. Thinking about your physical well‑being over the years, how do you feel the incident has influenced any challenges or difficulties you face today?
3. How has the attack affected your emotional or psychological health over time?
4. In what ways do you feel the incident shaped your experience of aging?
5. Have new health issues emerged that you link to the attack or its aftermath?
6. How would you describe your current sense of independence or dependence in daily life?
7. What financial considerations influence the treatments or therapies you pursue?
8. How do you cover health expenses?
9. How has the attack affected your access to informal support (family, friends, neighbours)?
10. Do you feel your social circle has changed because of the health challenges you face?
11. How do you make sense of the difference between “surviving” and “recovering”?
12. What coping strategies—physical, emotional, or spiritual—have helped you over time?
13. Are there ways in which the attack still influences how you see your body today?
14. Looking back, what resources or supports do you wish had been available to you?
15. What advice would you offer another older woman navigating long‑term recovery after a similar experience?
